# Alterations in transcriptional responses associated with vascular aging

**DOI:** 10.1186/1476-9255-6-16

**Published:** 2009-05-21

**Authors:** Yumei Zhan, Lei Yuan, Peter Oettgen

**Affiliations:** 1 Division of Cardiology, and Molecular and Vascular Biology, Department of Medicine, Center for Vascular Biology Research, Beth Israel Deaconess Medical Center, Boston, Massachusetts, USA

## Abstract

Vascular aging is an independent risk factor for cardiovascular disease that can occur in the absence of other traditional risk factors. Inflammation is a hallmark of vascular aging that ultimately leads to structural changes in the vessel wall including an increase in medial thickness and perivascular fibrosis. Several classes of transcription factors have been identified that participate in the regulation of cellular responses associated with vascular aging. Nuclear factor (NF)-κB is the prototypic example of a transcriptional activator in the setting of inflammation, being activated in response to multiple inflammatory mediators including pro-inflammatory cytokines and bacterial endotoxin. In contrast, the activation of the nuclear hormone receptor and transcription factor peroxisome proliferator-activated receptor-alpha (PPAR-α) results in its translocation from the cell surface to the nucleus where it exerts anti-inflammatory effects. Vascular aging is also associated with endothelial dysfunction. One important repair mechanism for improving endothelial function is the recruitment of endothelial progenitor cells (EPCs). In the setting of aging the number of EPCs diminishes which has been linked to a decrease in the activity and/or expression of the transcription factor hypoxia inducible factor (HIF)-1 alpha. A change in the balance of the activity of pro-inflammatory transcription factors versus those that inhibit inflammation likely contributes to the process of vascular aging. The purpose of this review is to summarize our current knowledge of these age-related changes in transcriptional responses, and to discuss the therapeutic potential of targeting some of these factors.

## Arterial aging

Epidemiological studies strongly support that vascular aging, which is accompanied by increased arterial stiffness, is an independent risk factor for cardiovascular morbidity and mortality [1-4]. Arterial stiffness is also frequently associated with the presence or the development of hypertension [5,6]. Whether an increase in arterial stiffness always precedes the onset of hypertension has not been determined. Although the precise molecular mechanisms underlying arterial aging have not been elucidated, inflammation appears to be a central component [7-9]. Some of the mediators of this inflammatory response include pro-inflammatory cytokines such as tumor necrosis alpha (TNF-α), transforming growth factor-beta (TGF-β), and angiotensin II (Ang II). The focus of this article is to review some of the transcriptional mediators that are responsible for activating critical genes involved in the initiation and propagation of vascular aging (Table 1).

**Table 1:** Roles of selected transcription factors in vascular inflammation and aging

Transcription Factors	Pro-/Anti-inflammatory	Selected Target Genes	Alterations with Aging
NF-κB	Pro-inflammatory	MCP-1, ICAM-1, NOS2	Increased

HIF-1α	Anti-inflammatory	VEGF, SDF-1	Decreased

Ets-1	Pro-inflammation	MCP-1, VCAM-1, PDGF, p16^INK4a^	Increased

ERG	Anti-inflammatory	VWF, VE-Cad, Ang2, IL-8	Unknown

PPAR-α, -γ	Pro-inflammatory	NF-|B, pro-inflammatory cytokines, NOS2, VCAM-1	Decreased

## NF-κB

NF-κB is known as the prototypic transcriptional mediator of inflammation. Under non-inflammatory conditions the heterdimeric Rel domain subunits, p50 and p65, of NF-κB are constitutively expressed but remain inactivated in the cytoplasm bound to the inhibitory protein Iκ-B. In response to inflammation Iκ-B is degraded allowing p50 and p65 to form NF-κB that can now freely translocate to the nucleus [10]. It was recently demonstrated that not only the expression but the activity of NF-κB is up-regulated during the process of aging [11,12]. The expression and activity of NF-κB was evaluated in fibroblasts from patients with ages ranging from 22 to 92 years of age. Over time there was a significant increase in the activity of NF-κB and the expression of inflammatory genes.

During the process of vascular aging there is a similar increase in NF-κB expression and activity in vascular smooth muscle cells and endothelial cells. This has been attributed to a variety of different mechanisms [13]. First, there is an increase in the levels of circulating cytokines, and in particular TNF-α during the process of aging. Second, vascular aging is associated with the increased production of reactive oxygen species (ROS), and in particular mitochondrial-derived H_2_O_2 _due to age-related mitochondrial dysfunction [12,14,15]. In the vascular endothelium age related increases in the expression of NF-κB are associated with increased expression of monocyte chemoattractant protein 1 (MCP-1) and a reduction in endothelium-dependent dilation [16]. Similarly the activity and response of NF-κB to pro-inflammatory cytokines was enhanced in aged compared to young vascular smooth muscle cells and was associated with an augmentation of the induction of ICAM-1 and inducible nitric oxide synthase (NOS2) genes [17].

## Hypoxia Inducible Factor-1 alpha (HIF-1α)

HIF-1α is a member of the transcription factor family and has been shown to be a critical regulator of neovascularization [18]. HIF-1α is activated in the setting of hypoxia and promotes the expression of vascular endothelial growth factor (VEGF) [19]. In the setting of myocardial ischemia or infarction the activation of HIF-1α promotes local angiogenesis through the expression of VEGF. In addition HIF-1α can induce the expression of stromal cell-derived factor-1 (SDF-1) [20]. SDF-1 enhances the recruitment of endothelial progenitor cells (EPCs) in the setting of tissue injury or ischemia. One of the hallmarks of vascular aging is endothelial dysfunction. EPCs can promote the repair of dysfunctional or damaged endothelium. Recent studies suggest that the levels and activity of HIF-1α diminish with aging and thereby leads to reduced levels of SDF-1 [21,22]. The results of these studies suggest that both local proliferation of endothelial cells by VEGF in the setting of ischemia, and the recruitment of EPCs to promote neovascularization or repair damaged endothelium are diminished with aging.

## ETS factor family

The ETS factors are a family of transcription factors that share a highly conserved DNA binding domain (Ets domain). ETS factors are involved in regulating a wide variety of biological processes including normal development and differentiation [23]. Until recently, very little was known about a role for ETS factors in regulating vascular inflammation. Over the past few years several studies have been completed that support a role for several ETS family members in the regulation of vascular inflammation, including endothelial activation in response to inflammatory mediators, the recruitment of inflammatory cells to the vessel wall, and proliferation and migration of vascular smooth muscle cells. We and others have observed that Ets-1 is induced in VSMC and endothelial cells in response to a variety of stimuli including Angiotensin II (Ang II), PDGF-BB, thrombin, interleukin-1 beta (IL-1β), and tumor necrosis alpha (TNF-α) [24-30]. Target genes identified to be downstream of Ets-1 in the setting of acute vascular inflammation include the chemokine MCP-1 and the adhesion molecule VCAM-1. Systemic administration of the vasoactive peptide Ang II via continuous infusion is not only associated with increases in blood pressure but also promotes the recruitment of inflammatory cells, including T cells and monocytic cells, to the vessel wall. The influx of inflammatory cells in response to Ang II is markedly diminished in Ets-1 deficient mice compared to littermate controls[27]. One of the major mediators of vascular inflammation within the vessel wall is ROS. Ang II, for example, promotes the generation of superoxide anions in VSMC largely via the activity of NADPH oxidases, that can be converted to hydrogen peroxide by superoxide dismutase [31]. Reactive oxygen species, and in particular hydrogen peroxide, can also stimulate Ets-1 expression [32]. Ets-1 functions synergistically with the transcription factor Sp1 to regulate the expression of the PDGF receptor in an ROS-dependent manner. Ets-1 and Sp1 are enriched in VSMC found in human atherosclerotic lesions that express increased levels of the PDGF receptor.

The tumor suppressor molecule p16^INK4a ^is a principal mediator of cellular senescence [33,34]. Increased levels of p16^INK4a ^have been detected in a number of different cell types associated with aging including vascular smooth muscle cells [35]. The molecular mechanisms by which p16^INK4a ^is regulated have not been fully elucidated, however at the transcriptional level it has recently been shown that Ets-1 is a critical factor in determining expression levels of p16^INK4a ^in a number of cells and tissues during the process of aging [36]. The age related increases in the expression of Ets-1 and p16^INK4a ^are diminished by caloric restriction that is associated with weight gain. Administration of resveratrol, a natural atoxic phytoestrogen, to mice, mimics the transcriptional effects of caloric restriction [37]. Resveratrol is an activator of sirtuins (SIRT1). Sirtuins are a family of NAD^+^-dependent deacetylases that can inhibit cell senescence. Resveratrol has been shown to reduce the levels of p16^INK4a ^through activation of SIRT1 [38]. The administration of resveratrol to mice, prevented age related reductions in endothelial function [37]. Resveratrol is a potent inhibitor of NF-κB activation in endothelial cells [39]. Similarly in obese mice, that exhibit a more rapid decline in vascular function that is associated with a pro-inflammatory state, administration of resveratrol reduced the obesity related endothelial dysfunction, that was at least in part related to a reduction in the generation of ROS [37].

ERG is an ETS family member that has been shown to contribute to the regulation of a number of endothelial-restricted genes including VE-cadherin, vWF, and angiopoietin-2 [40-42]. ERG is markedly downregulated in human endothelial cells in response to TNF-α. We have recently demonstrated that ERG functions as a suppressor of EC activation [43]. Suppression of ERG using siRNA results in an increase in neutrophil attachment that is dependent on increased expression of interleukin-8 by endothelial cells. A significant number of genes that are up or down regulated by ERG suppression in endothelial cells overlap with genes that are similarly up or down regulated by TNF-α.

## PPAR family

More recently selected transcription factors have been identified that exhibit anti-inflammatory properties and can modulate the initial cascade of genes induced in response to inflammatory stimuli. For example, the PPAR (peroxisome proliferators-activated receptors) nuclear receptors are transcription factors expressed in EC, VSMC, and monocytic cells. Activation of PPARα and PPARγ receptors are associated with favorable effects on lipid metabolism and insulin sensitivity that are also beneficial with regard to limiting the development of atherosclerosis [44]. Binding of PPAR agonists to their cognate receptors is also associated with anti-inflammatory effects. Activation of the PPARγ pathway, for example, can inhibit the activity of the transcription factors AP-1 and NF-κB in response to pro-inflammatory cytokines such as TNF-α in endothelial cells [45]. Activation of PPARγ also inhibits the process of vascular aging in rats [46,47]. For example, the administration of the PPARγ agonist 2,4-thiazolidinedione (2,4-TZD) to rats of varying ages was associated with a reduction in the activity of NF-κB, pro-inflammatory cytokines, NOS2, and vascular cell adhesion molecule-1 (VCAM-1) in the kidney. The upregulation of NF-κB and associated inflammatory genes in the absence of treatment is an aged related phenomenon.

## Targeting transcription factors

The elucidation of the critical transcriptional factors that regulate vascular inflammation may therefore not only advance our basic understanding of the molecular mechanisms of vascular inflammation but may also provide novel therapeutic targets for drug discovery. Historically, transcription factors have not been viewed as good targets for drug therapy, with the exception of nuclear hormone receptors that often reside on the cell surface and are activated by ligands that promote their transfer into nucleus where they function as transcription factors and bind to specific gene targets. One approach that has been used to target transcription factors in vivo is through the development of membrane permeable peptides that can competitively inhibit the binding of the transcription factors to the DNA. This approach was used to block the function of the ETS factor ELF-1 to inhibit the expression of the endothelial restricted genes Tie2 and endothelial nitric oxide synthase (eNOS) and block tumor angiogenesis in vivo [48]. A similar approach was used in vivo to block the activity of Ets-1 and inhibit the generation of ROS, induction of inflammatory genes, and favorably effect vascular remodeling in response to Ang II infusion in mice over two weeks [49]. The ability to identify small molecules that specifically block transcription factors that are not ligand-dependent has recently demonstrated [50]. Although only a few transcription factors have been targeted in this way, and no drugs, with the exception of those targeting the nuclear hormone receptors, are currently available to block these factors, several companies are actively pursuing these factors as therapeutic targets.

## Competing interests

The authors declare that they have no competing interests.

## Authors' contributions

YZ contributed to the writing of this manuscript. LJ contributed to the writing of this manuscript. PO contributed to the writing of this manuscript. All authors read and approved the final manuscript.
